# Supplementary material for: CryoSift – An accessible and automated CNN-driven tool for cryo-EM 2D class selection
Source: bioRxiv. 2025 Sep 10:2025.07.28.667259. Originally published 2025 Aug 1. Preprint. [Version 2] doi: 10.1101/2025.07.28.667259 (PMC12324380; doi:10.1101/2025.07.28.667259)
Supplement: Supplement 1 [file NIHPP2025.07.28.667259v2-supplement-1.pdf]

bioRxiv preprint doi: https://doi.org/10.1101/2025.07.28.667259; this version posted September 10, 2025. The copyright holder for this preprint (which was not certified by peer review) is the author/funder, who has granted bioRxiv a license to display the preprint in perpetuity. It is made available under aCC-BY 4.0 International license.

Supporting Information

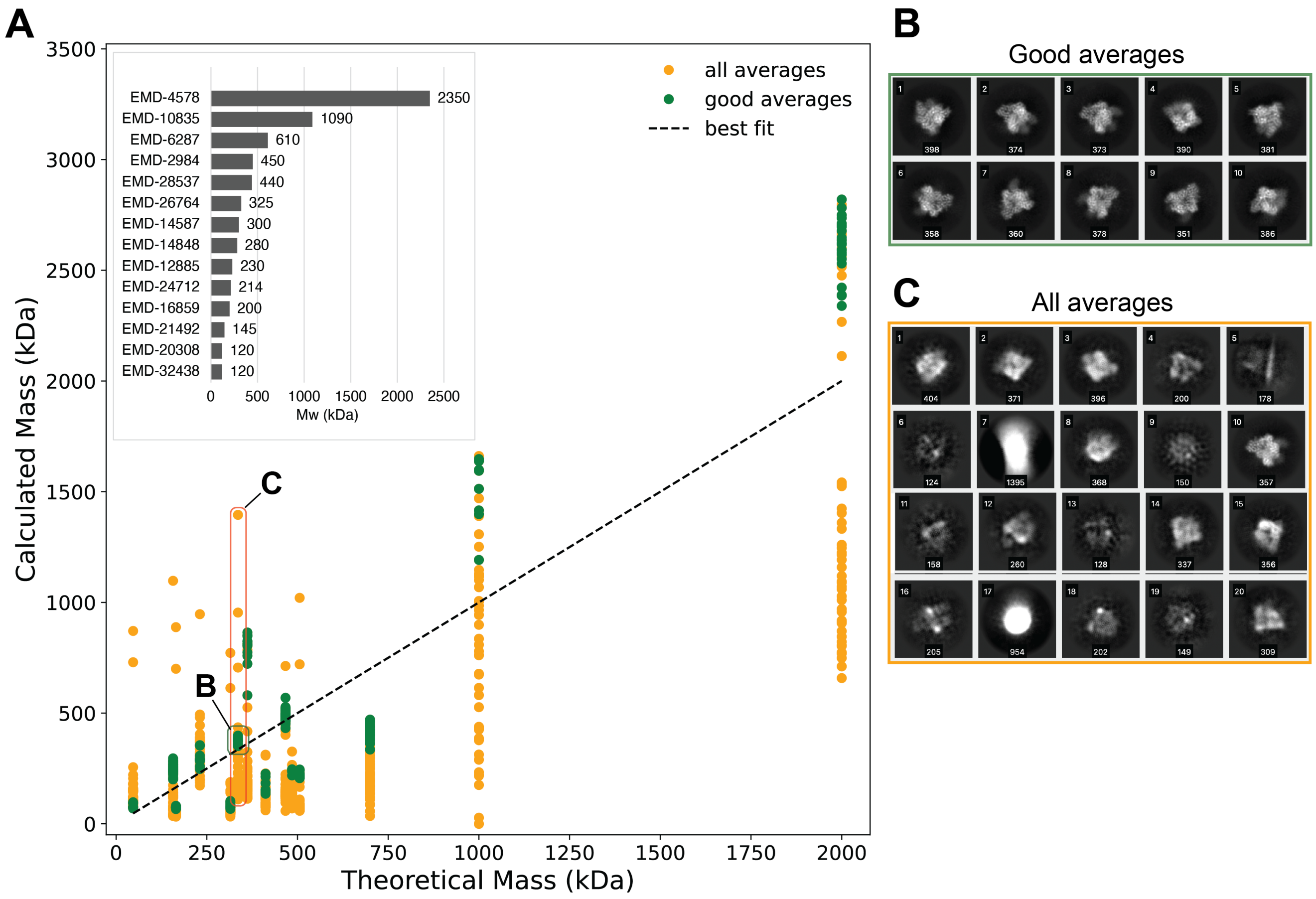

Figure S1. Mass Estimator. (**A**) Correlation of the calculated mass over theoretical mass (in kDa). All averages in orange and good averages in green. Linear fit as dashed lines. Inlet shows the utilized EMD entries sorted by molecular weight. (**B**) Examples of good 2D class averages with calculated mass labels. (**C**) range of all classes in selected example with calculated mass labels.

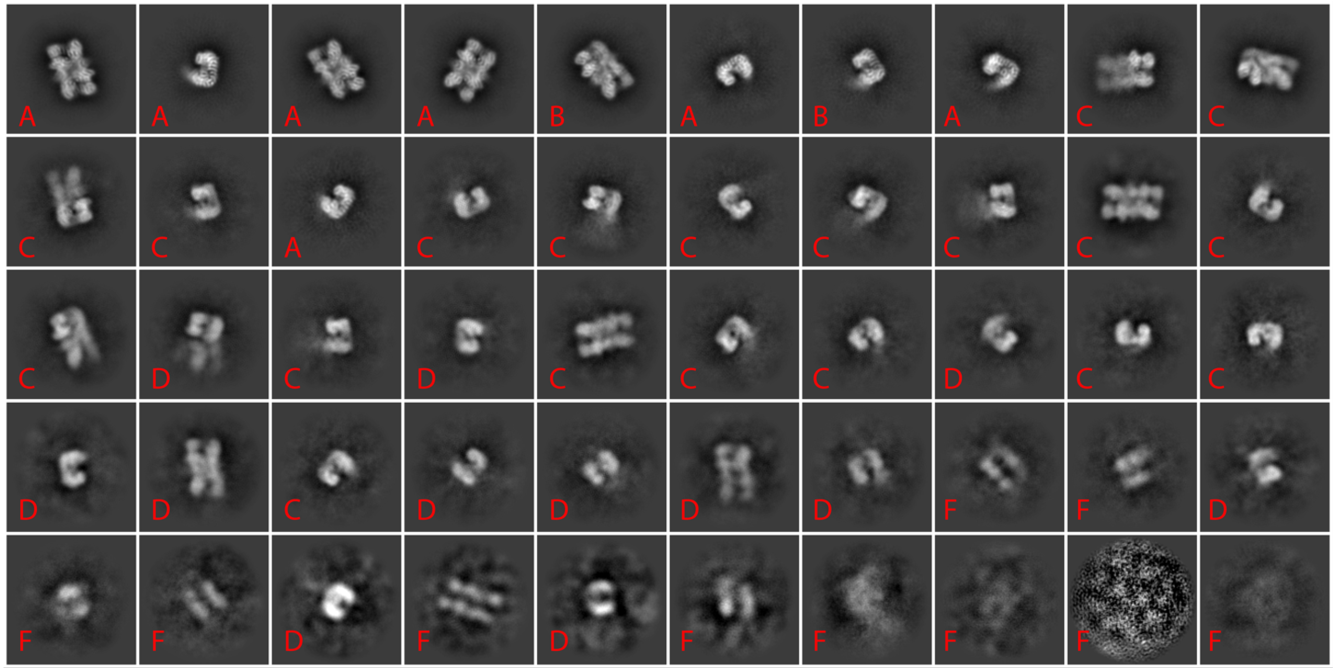

Figure S2. Example labeled class averages included in the grading rubric for assessors.

bioRxiv preprint doi: https://doi.org/10.1101/2025.07.28.667259; this version posted September 10, 2025. The copyright holder for this preprint (which was not certified by peer review) is the author/funder, who has granted bioRxiv a license to display the preprint in perpetuity. It is made available under aCC-BY 4.0 International license.

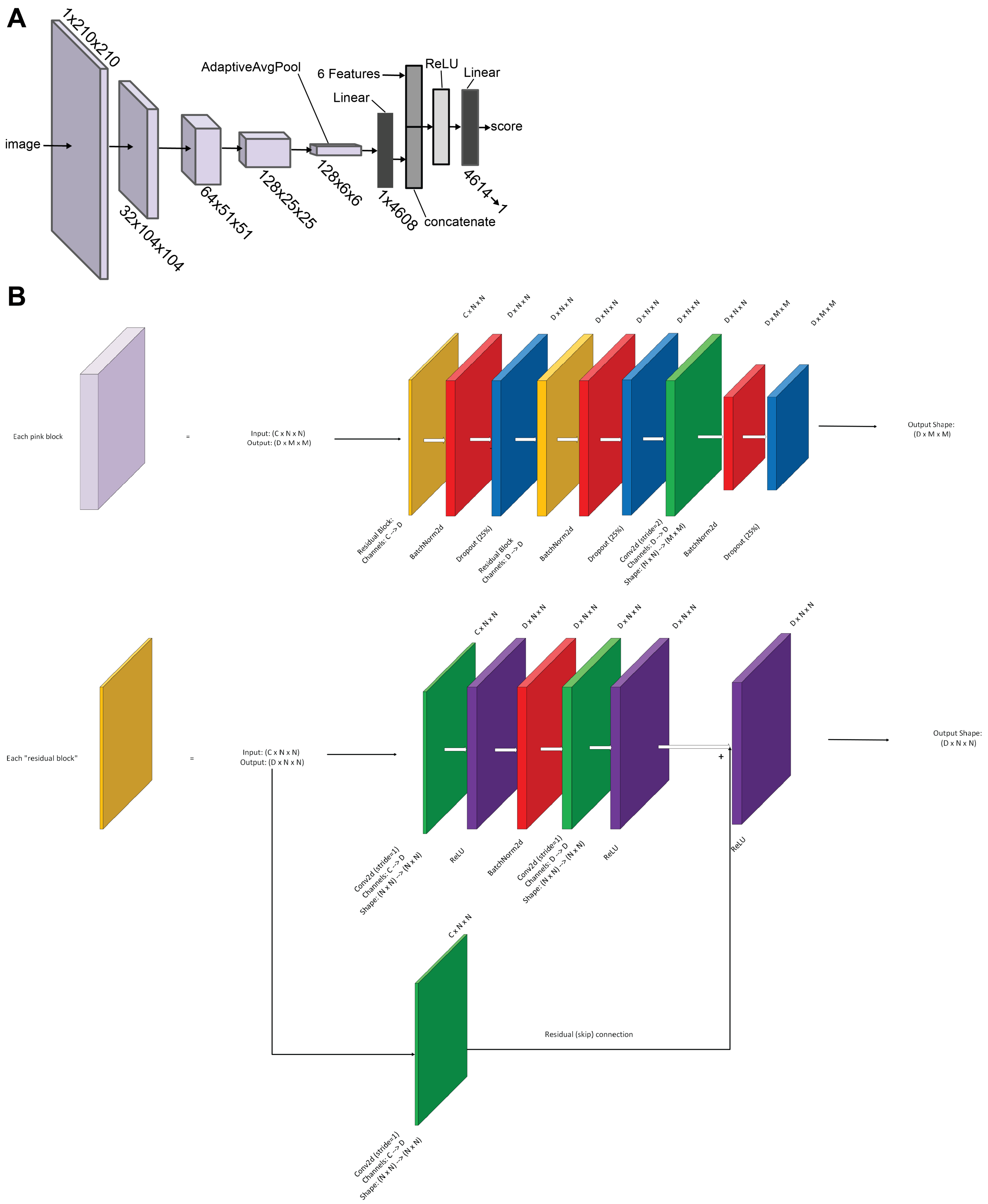

Figure S3. Detailed CNN design architecture. (**A**) Overview of the base layers. (**B**) Detailed layer contents and operations for main layers and each "residual block".

bioRxiv preprint doi: https://doi.org/10.1101/2025.07.28.667259; this version posted September 10, 2025. The copyright holder for this preprint (which was not certified by peer review) is the author/funder, who has granted bioRxiv a license to display the preprint in perpetuity. It is made available under aCC-BY 4.0 International license.

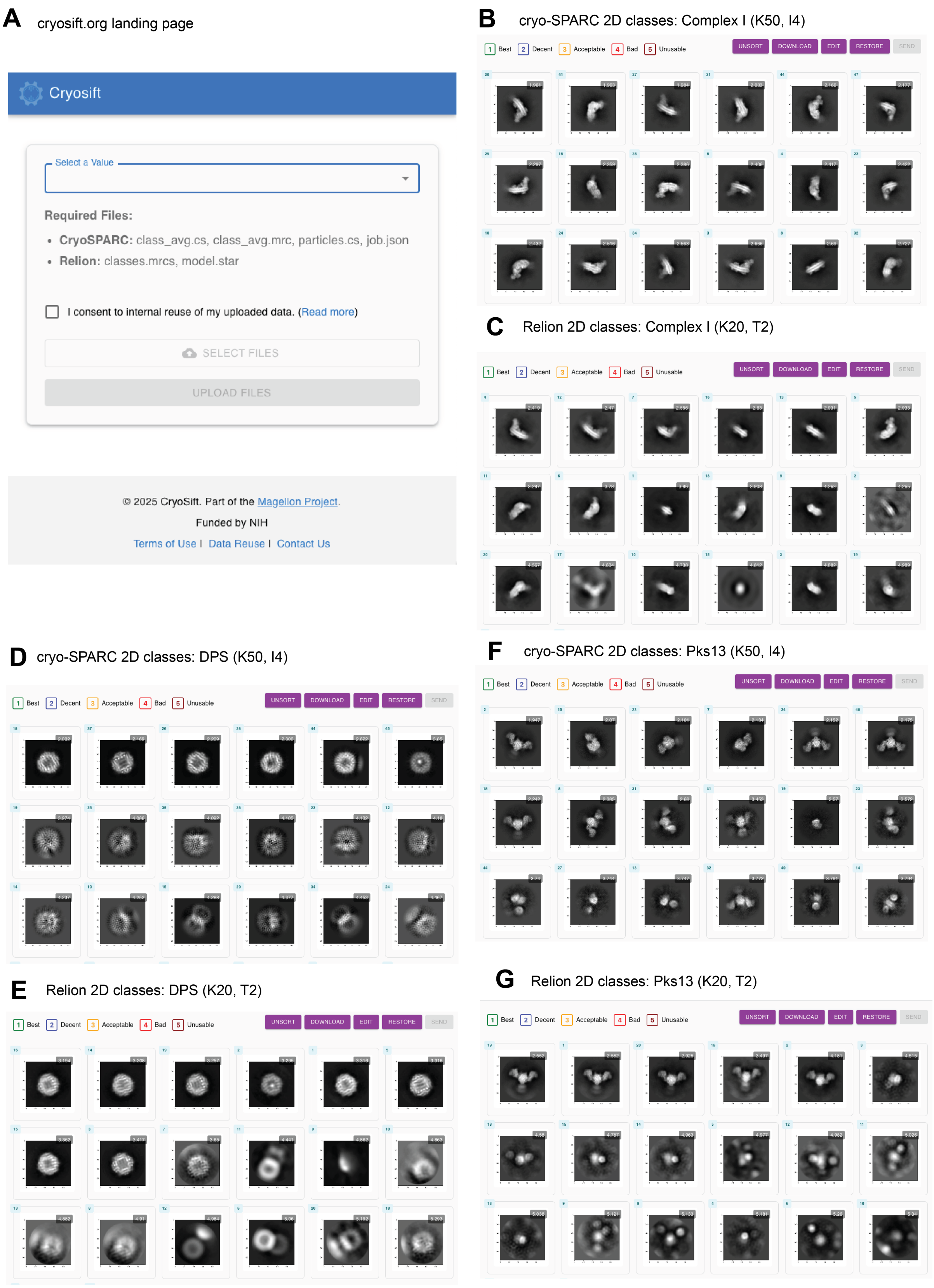

Figure S4. Preview of the CryoSift GUI available at https://www.cryosift.org. (**A**) Landing page. (**B**) Output of sorted Complex-I 2D classes from cryo-SPARC uploads (K50, 50 classes and I4, initial class uncertainty factor 4). (**C**) Output of sorted Complex-I 2D classes from RELION5 (VDAM, K50, T2). RELION re-processing from a re-extracted particle-stack using pyem for file conversion from cryo-SPARC.

bioRxiv preprint doi: https://doi.org/10.1101/2025.07.28.667259; this version posted September 10, 2025. The copyright holder for this preprint (which was not certified by peer review) is the author/funder, who has granted bioRxiv a license to display the preprint in perpetuity. It is made available under aCC-BY 4.0 International license.

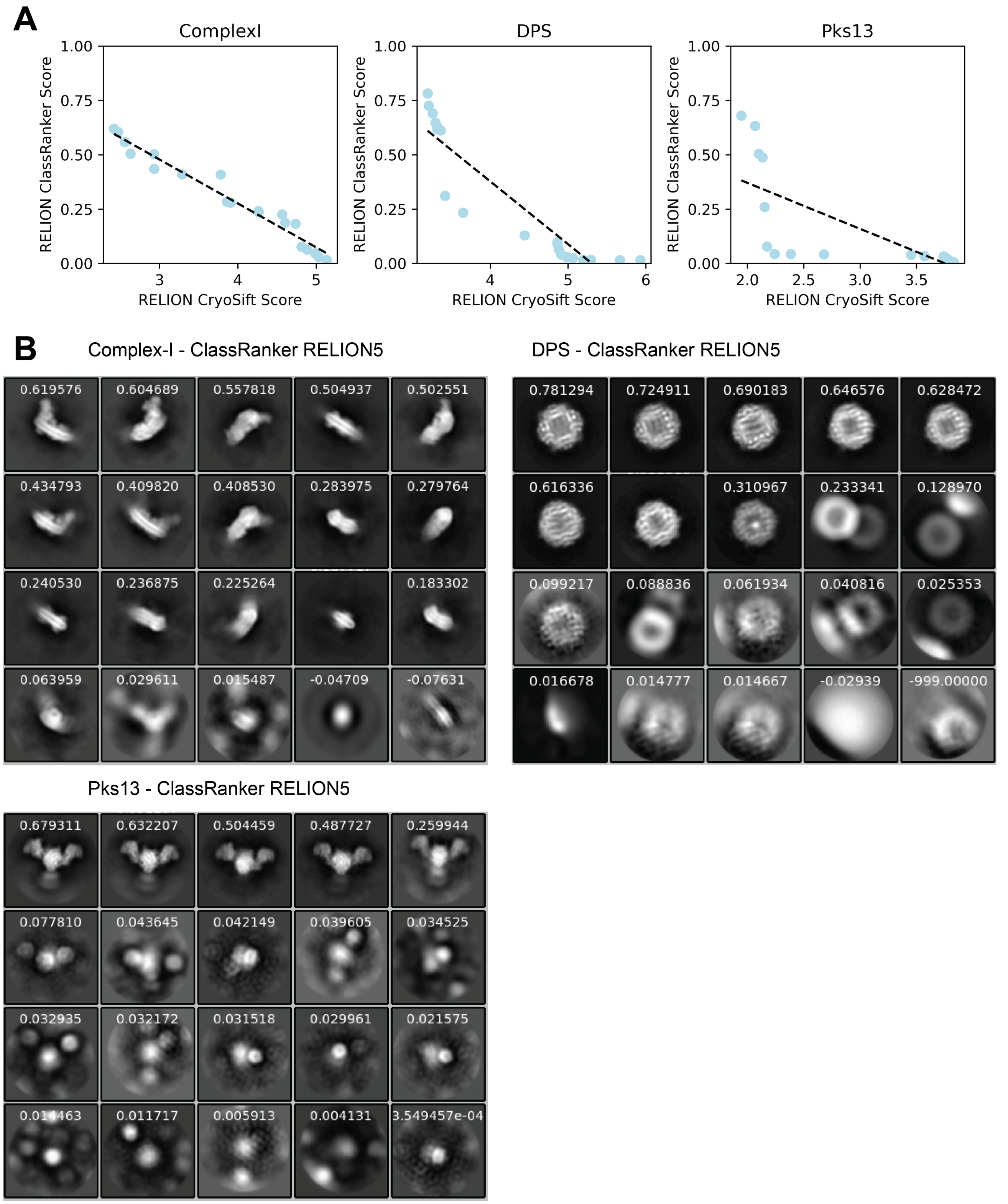

Figure S5. Comparison of labeling 2D classes. (A) Scotter plot and linear fit of RELION 5 processed datasets, showing it's native ClassRanker scored classes over corresponding CryoSift scored classes. (B) 2D class averages and ClassRanker scores used in (B). 2D averages and CryoSift scores used for (B) shown in Fig. S4.

bioRxiv preprint doi: https://doi.org/10.1101/2025.07.28.667259; this version posted September 10, 2025. The copyright holder for this preprint (which was not certified by peer review) is the author/funder, who has granted bioRxiv a license to display the preprint in perpetuity. It is made available under aCC-BY 4.0 International license.

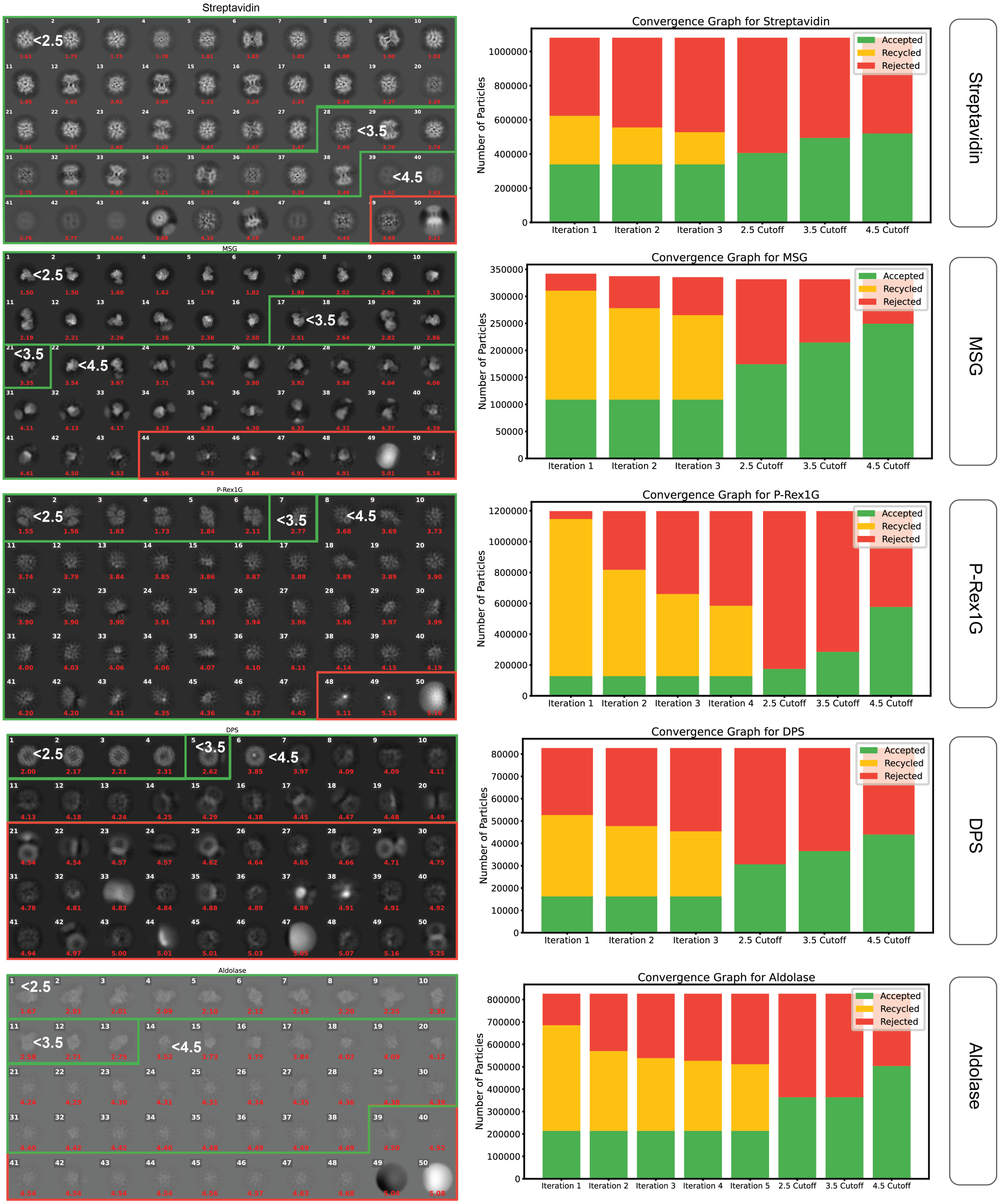

Figure S6. Labeled 2D averages of the last 2D classification for all test-datasets ordered by increasing CryoSift score (red) with associated convergence graphs for all iterations. Projections of matching cutoffs are grouped using green (accepted) and red (rejected) boxes using the respective threshold in white. For the convergence graphs, particle numbers were plotted over iterations. Accepted particles (green), recycled particles (yellow), and rejected particles (red). Additionally, the final number of accepted (green) and rejected (red) particles per CryoSift cutoff score (2.5, 3.5 and 4.5) are depicted.

bioRxiv preprint doi: https://doi.org/10.1101/2025.07.28.667259; this version posted September 10, 2025. The copyright holder for this preprint (which was not certified by peer review) is the author/funder, who has granted bioRxiv a license to display the preprint in perpetuity. It is made available under aCC-BY 4.0 International license.

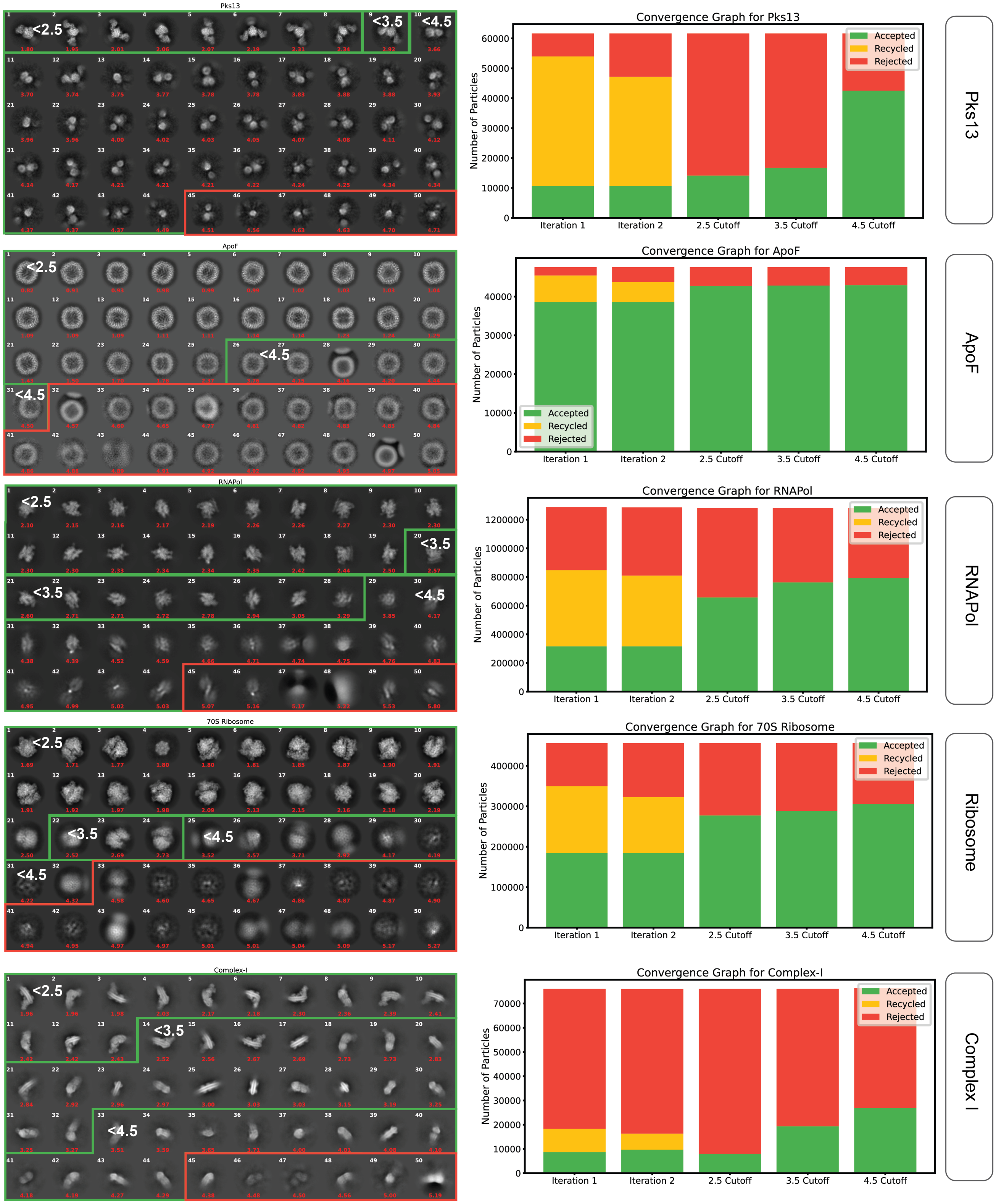

Figure S7. Continued
