# Supplementary material for: CryoSift – An accessible and automated CNN-driven tool for cryo-EM 2D class selection
Source: bioRxiv. 2025 Aug 1:2025.07.28.667259. Preprint. [Version 1] doi: 10.1101/2025.07.28.667259 (PMC12324380; doi:10.1101/2025.07.28.667259)
Supplement: Supplementary file 1 [file NIHPP2025.07.28.667259v1-supplement-1.pdf]

bioRxiv preprint doi: https://doi.org/10.1101/2025.07.28.667259; this version posted August 1, 2025. The copyright holder for this preprint (which was not certified by peer review) is the author/funder, who has granted bioRxiv a license to display the preprint in perpetuity. It is made available under aCC-BY 4.0 International license.

Supporting Information

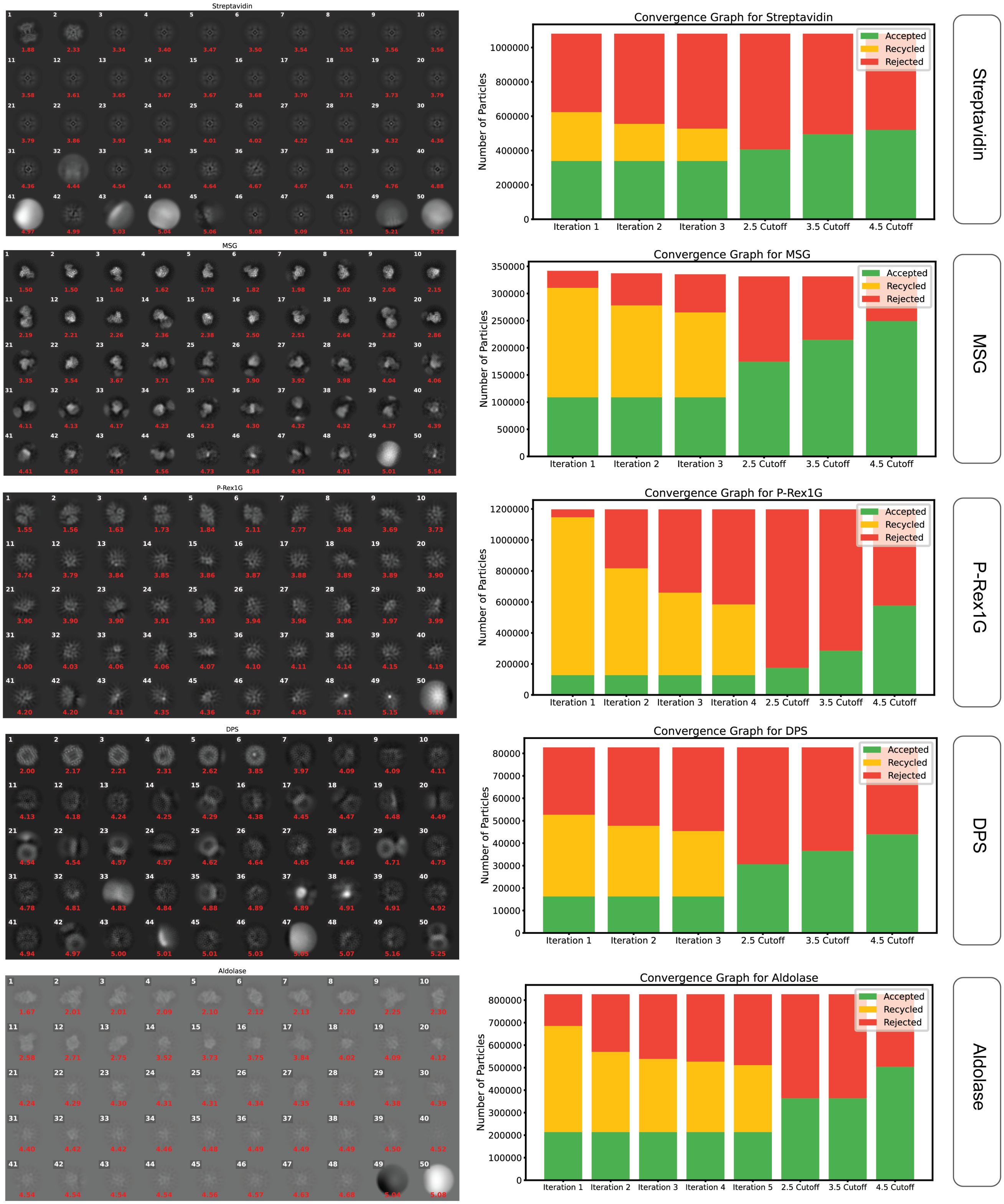

Figure S1. Labeled 2D averages of the first 2D classification for all test-datasets ordered by increasing 2D assess score (red) with associated convergence graphs for all iterations. For the convergence graphs, particle numbers were plotted over iterations. Accepted particles (green), recycled particles (yellow), and rejected particles (red). Additionally, the final number of accepted (green) and rejected (red) particles per 2D assess cutoff score (2.5, 3.5 and 4.5) are depicted.

bioRxiv preprint doi: https://doi.org/10.1101/2025.07.28.667259; this version posted August 1, 2025. The copyright holder for this preprint (which was not certified by peer review) is the author/funder, who has granted bioRxiv a license to display the preprint in perpetuity. It is made available under aCC-BY 4.0 International license.

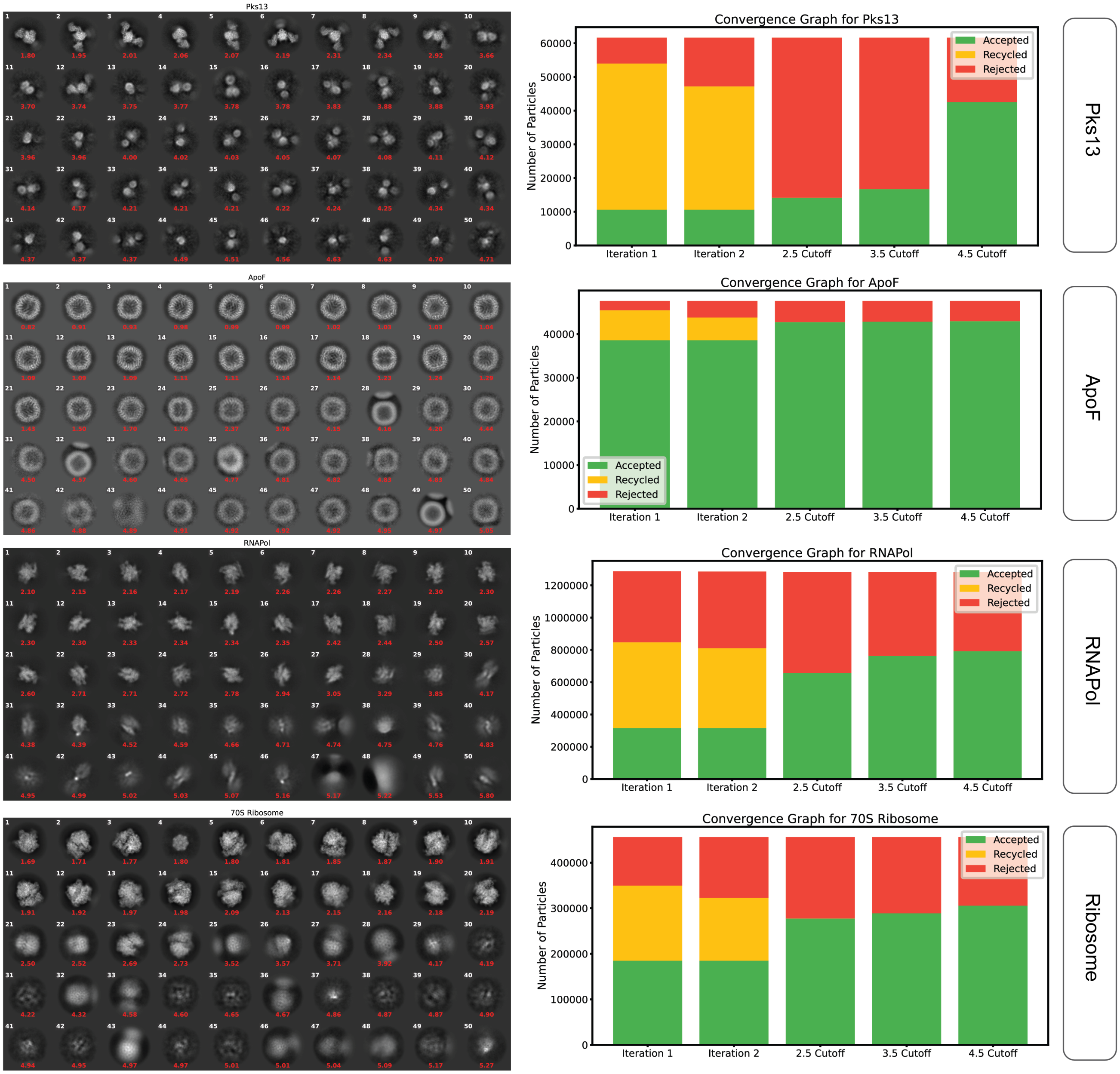

Figure S2. Continued

bioRxiv preprint doi: https://doi.org/10.1101/2025.07.28.667259; this version posted August 1, 2025. The copyright holder for this preprint (which was not certified by peer review) is the author/funder, who has granted bioRxiv a license to display the preprint in perpetuity. It is made available under aCC-BY 4.0 International license.

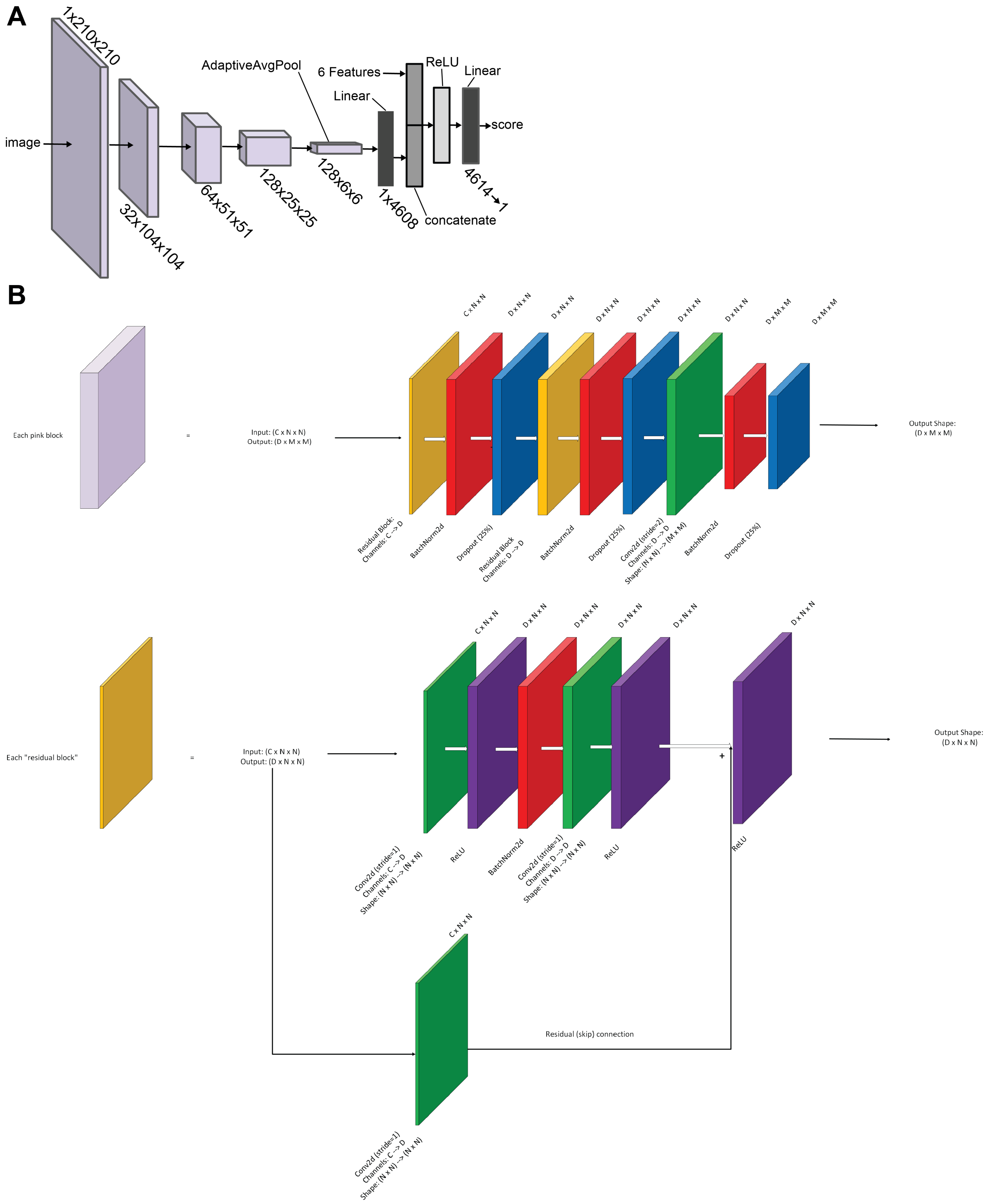

Figure S3. Detailed CNN design architecture. (**A**) Overview of the base layers. (**B**) Detailed layer contents and operations for main layers and each "residual block".

bioRxiv preprint doi: https://doi.org/10.1101/2025.07.28.667259; this version posted August 1, 2025. The copyright holder for this preprint (which was not certified by peer review) is the author/funder, who has granted bioRxiv a license to display the preprint in perpetuity. It is made available under aCC-BY 4.0 International license.

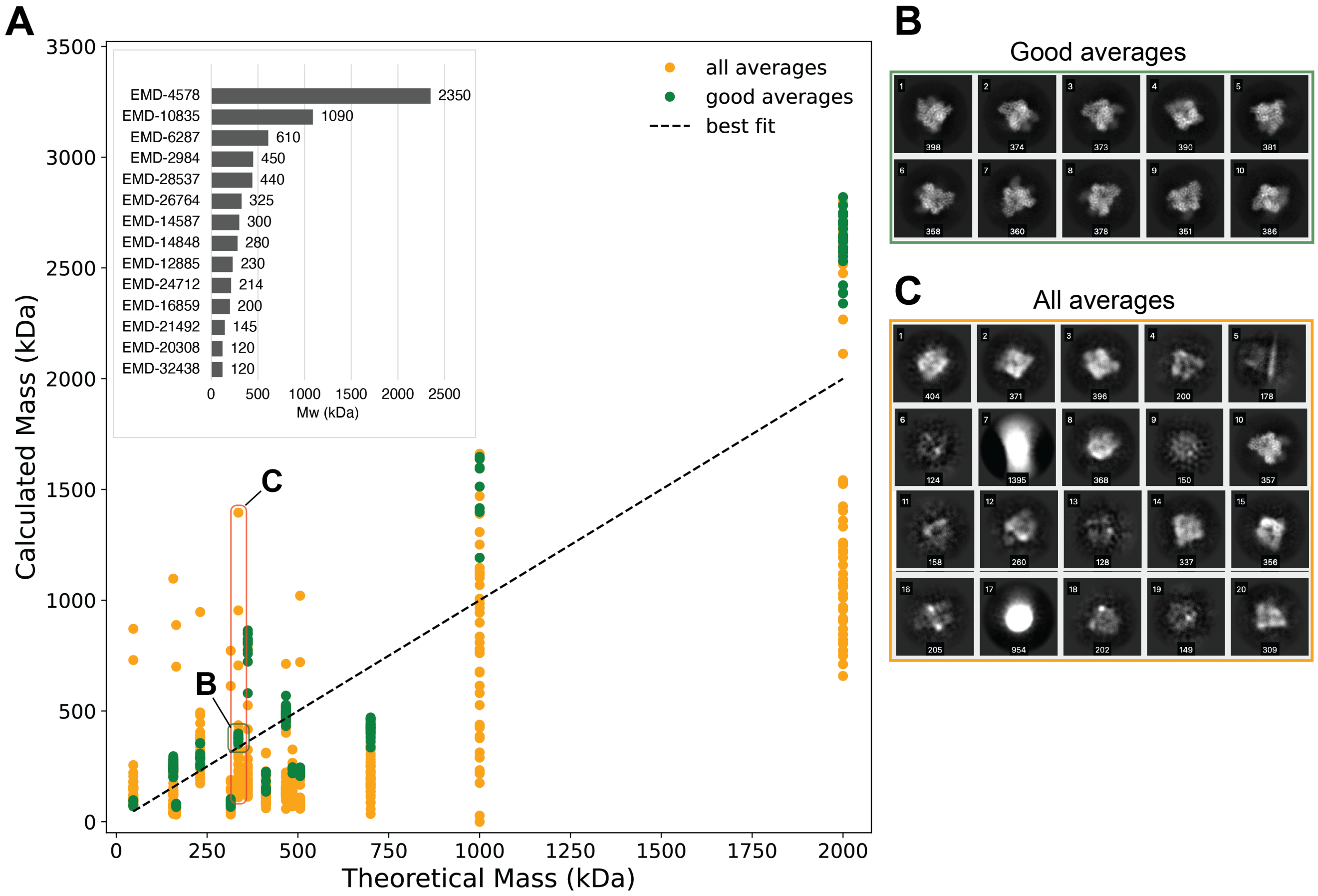

Figure S4. Mass Estimator. (**A**) Correlation of the calculated mass over theoretical mass (in kDa). All averages in orange and good averages in green. Linear fit as dashed lines. Inlet shows the utilized EMD entries sorted by molecular weight. (**B**) Examples of good 2D class averages with calculated mass labels. (**C**) range of all classes in selected example with calculated mass labels.

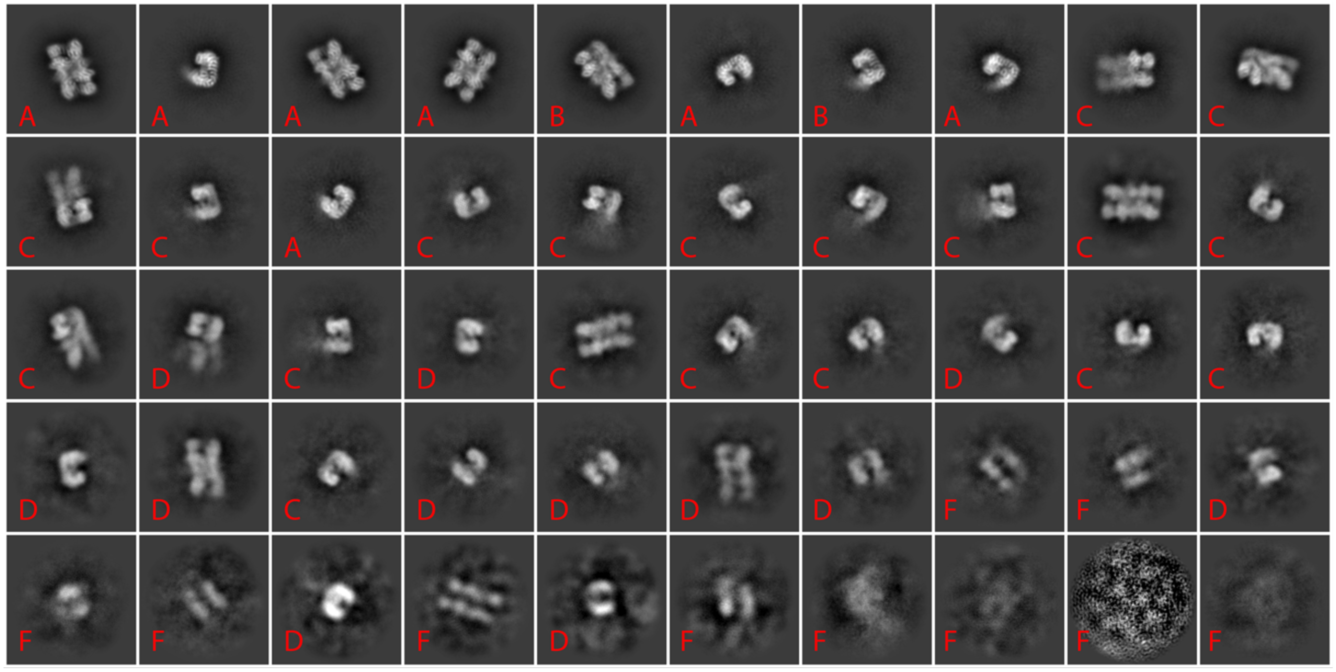

Figure S5. Example labeled class averages included in the grading rubric for assessors.

bioRxiv preprint doi: https://doi.org/10.1101/2025.07.28.667259; this version posted August 1, 2025. The copyright holder for this preprint (which was not certified by peer review) is the author/funder, who has granted bioRxiv a license to display the preprint in perpetuity. It is made available under aCC-BY 4.0 International license.

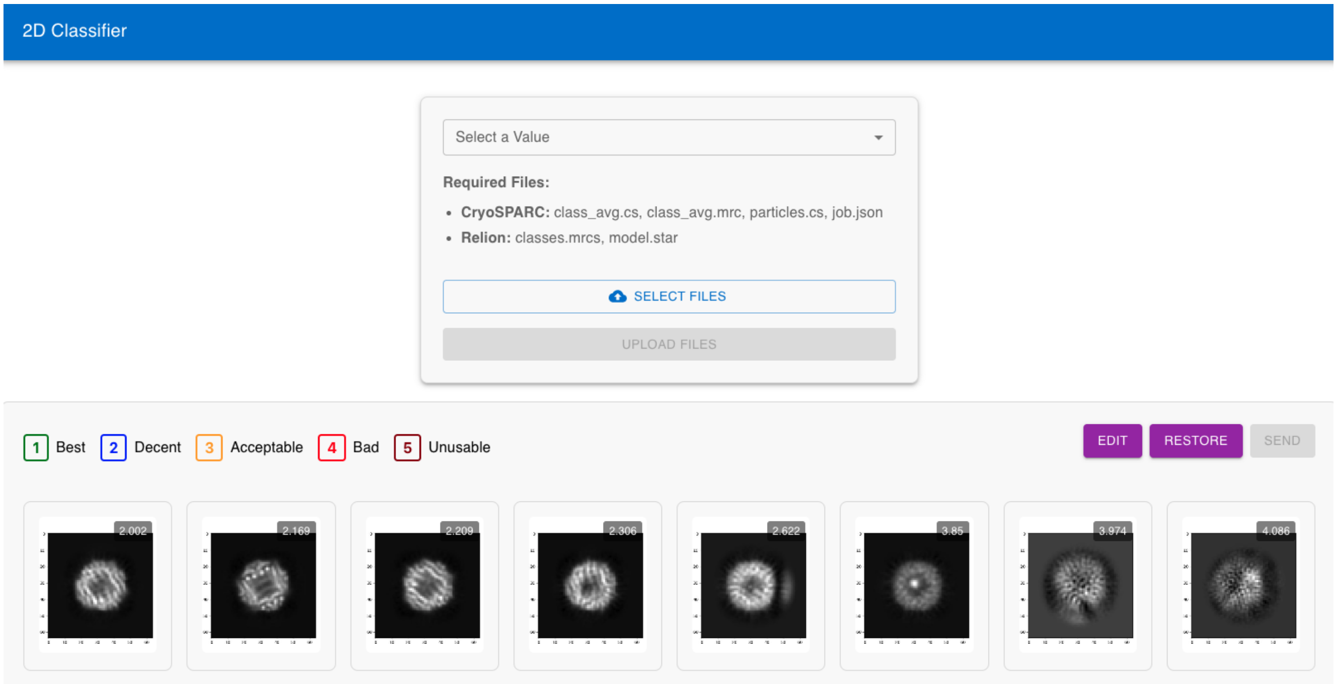

Figure S6. Preview of the CryoSift GUI available at `https://www.cryosift.org`.
